# Advancements in therapeutically targeting orphan GPCRs

**DOI:** 10.3389/fphar.2015.00100

**Published:** 2015-05-08

**Authors:** Jennifer A. Stockert, Lakshmi A. Devi

**Affiliations:** Department of Pharmacology and Systems Therapeutics, Icahn School of Medicine at Mount Sinai, New York, NYUSA

**Keywords:** Homology modeling, virtual screening, MD simulations, protein crystallography

## Abstract

G-protein coupled receptors (GPCRs) are popular biological targets for drug discovery and development. To date there are more than 140 orphan GPCRs, i.e., receptors whose endogenous ligands are unknown. Traditionally orphan GPCRs have been difficult to study and the development of therapeutic compounds targeting these receptors has been extremely slow although these GPCRs are considered important targets based on their distribution and behavioral phenotype as revealed by animals lacking the receptor. Recent advances in several methods used to study orphan receptors, including protein crystallography and homology modeling are likely to be useful in the identification of therapeutics targeting these receptors. In the past 13 years, over a dozen different Class A GPCRs have been crystallized; this trend is exciting, since homology modeling of GPCRs has previously been limited by the availability of solved structures. As the number of solved GPCR structures continues to grow so does the number of templates that can be used to generate increasingly accurate models of phylogenetically related orphan GPCRs. The availability of solved structures along with the advances in using multiple templates to build models (in combination with molecular dynamics simulations that reveal structural information not provided by crystallographic data and methods for modeling hard-to-predict flexible loop regions) have improved the quality of GPCR homology models. This, in turn, has improved the success rates of virtual ligand screens that use homology models to identify potential receptor binding compounds. Experimental testing of the predicted hits and validation using traditional GPCR pharmacological approaches can be used to drive ligand-based efforts to probe orphan receptor biology as well as to define the chemotypes and chemical scaffolds important for binding. As a result of these advances, orphan GPCRs are emerging from relative obscurity as a new class of drug targets.

## Introduction

G-protein coupled receptors are by far the largest group of TM signaling receptors in the human genome. Phylogenetic analysis has estimated that over 800 genes encode for GPCRs (Fredriksson et al., 2003), which amounts to over 1% of the entire human genome (Jassal et al., 2010). GPCRs are ubiquitously expressed throughout the body and modulate a wide array of essential physiological processes, including those governing the senses of vision, taste, and olfaction, as well as mediating cellular responses to hormones and neurotransmitters (Rosenbaum et al., 2009). GPCR signal transduction is accomplished by the binding of a receptor agonist to an exposed extracellular or intramembrane site on the receptor, which causes a conformational change in the receptor protein. This leads to the uncoupling of the heterotrimeric G-proteins associated with the receptor which, in turn, unleashes a cascade of intracellular downstream effector molecules. The assortment of known GPCR ligands is as expansive and diverse as the biological functions that they regulate, and includes photons, ions, odorants, amino acids, peptides, nucleotides, lipids, and small organic molecules (Kroeze et al., 2003; Venkatakrishnan et al., 2013). Because of the range of biological processes, and therefore pathologies, that they modulate, GPCRs have been popular and successful biological targets in the history of drug development.

Despite the diversity of this superfamily of proteins, all GPCRs have the same basic structure and signaling mechanisms. Thus the structure of a GPCR can be broken down into three basic parts: (i) the extracellular-facing region comprising the N-terminus and three ECL (ECL1–ECL3), (ii) the TM region, a defining motif of GPCRs, that consists of seven α-helices (labeled TM1–TM7) bundled together and which span the width of the membrane at varying lengths and angles, and (iii) the intracellular region that contains three ICL (ICL1–ICL3), an additional helix (H8) and C-tail. While the extracellular domain mediates the access of ligand(s) to the receptor (Venkatakrishnan et al., 2013), the TM region undergoes a conformational change upon the binding of a ligand that is transmitted to the intracellular region of the GPCR (Venkatakrishnan et al., 2013). The latter region is responsible for interacting with downstream effectors such as G-proteins (i.e., Gα and Gβγ subunits) and arrestins (Katritch et al., 2012). In canonical GPCR signaling, agonist binding to the receptor leads to the activation of G-proteins and these, in turn, activate effector molecules such as adenylyl cyclase and PLCβ resulting in the production of second messengers such as cAMP, DAG and Ins(1,4,5)P3, that continue the signaling cascade by activating further downstream proteins including PKA and PKC (Ritter and Hall, 2009), to name a few.

Given the size and scope of the GPCR superfamily, several classification schemes have been proposed. While some of these attempted to group GPCRs based on either the dynamics of ligand binding, physiological, or structural characteristics, the best-known classification scheme categorizes GPCRs based on their sequence and structural similarity (Fredriksson et al., 2003). Thus, phylogenetic analyses of non-olfactory human GPCRs have resulted in their grouping into five distinct classes abbreviated as GRAFS: glutamate (i.e., Family C receptors) rhodopsin (i.e., Family A receptors), adhesion, Frizzled/Taste2, and secretin (i.e., Family B receptors) receptors (Fredriksson et al., 2003; Rosenbaum et al., 2009; **Table 1**). Class A rhodopsin-like receptors form the largest, most diverse, and best studied, and contains over 700 receptors; ~240 members of this family are non-olfactory and include prostaglandin, amine (e.g., serotonin, dopamine, and histamine), and melatonin receptors (Fredriksson et al., 2003). The secretin and glutamate families both contain ~15 members and include the glucagon and GABA receptors, respectively. The adhesion family, so-called because they contain one or more domains with adhesion-like motifs at the N-terminus, comprises ~24 members, while the Frizzled/Taste2 family contains ~24 receptors that mediate cellular events in metazoan development and the sense of taste (Fredriksson et al., 2003).

**Table 1:** Solved structures of the GPCR families.

GPCR family	Identified member receptors	Solved receptor structures
Rhodopsin	701	21
Adhesion	24	0
Frizzled/Tas2	24	1
Glutamate	15	1
Secretin	15	2

*The largest GPCR family, Rhodopsin, has the largest number of solved structures. Since the first structure of a GPCR, bovine rhodopsin, was published in 2000, 24 other GPCR structures have been solved, indicative of the acceleration in solving GPCR structures.*

At present there are over 140 non-olfactory GPCRs for which endogenous ligands have not been identified or whose biological functions remain unknown (Tang et al., 2012). Conventionally it has been considered important to identify the endogenous ligand(s) activating a GPCR in order to study the receptor’s signaling/regulatory mechanisms and physiological function. Without this basic information, developing therapeutics targeting these receptors is considered to be nearly impossible (Yoshida et al., 2012). In the case of orphan GPCRs, the lack of knowledge about the endogenous ligand has focused research on the identification of cognate GPCR-ligand pairs rather than lead-compound identification and drug discovery.

Orphan receptors that have been matched to their endogenous ligand are termed deorphanized receptors. The earliest attempts to deorphanize GPCRs began in the latter half of the 1980s, and this coincided with advances in molecular biological techniques including low-stringency hybridization and degenerate polymerase chain reaction, which allowed for the successful cloning of orphan GPCRs (Civelli et al., 2013). Moreover, modern cloning technology allowed researchers to express orphan GPCRs in cell lines so that they could be tested in signal transduction assays with potential exogenous or endogenous ligands (Civelli et al., 2013). This strategy known as “reverse pharmacology” was used in the 1990s to deorphanize many ligand-receptor systems, including nociception for ORL-1, orexin-A for OX_1_R, orexin-B for OX_2_R, and ghrelin for GHS-R (Yoshida et al., 2012). The endogenous ligands were then used to further examine the pharmacological and physiological properties of the receptor *in vitro* or *in vivo* (Yoshida et al., 2012).

## Orphan GPCRs as Therapeutic Targets

Studies examining the distribution and localization as well as studies probing the behavioral phenotype of animals lacking the orphan GPCRs have been central to establishing the particular receptor as an attractive therapeutic target. Studies examining receptor expression by *in situ* hybridization and studies investigating the phenotypic characterization of targeted KD/KO of orphan GPCRs have proven extremely useful in elucidating their biological functions, and in suggesting their role as potential drug targets. For example, a study with the orphan receptor GPR88 used molecular and behavioral tests to propose a role for this receptor in schizophrenia (Logue et al., 2009). GPR88 mRNA was found to be highly expressed in the striatum of WT mice brains and absent in mice lacking GPR88 (GPR88 KO) using *in situ* hybridization (Logue et al., 2009). GPR88 KO mice had higher levels of phosphorylated DARPP-32 and increased sensitivity to dopamine, suggesting that GPR88 may play an important role in striatal function and dopamine response, making this orphan receptor a potential drug target for the treatment of psychiatric disorders involving the striatum like schizophrenia. Another study with the orphan GPR161 (also known as RE2) proposed a role for this receptor in the proper formation of the tubes of the heart (Leung et al., 2008). In this case *in situ* hybridization with developing embryos revealed GPR161 mRNA expression in the precardiac mesoderm, and knock-down of GPR161 resulted not only in pericardial edema, improper positioning of the ventricle and atrium, malformation of cardiac loops, and left–right (L–R) patterning, but also elevated Ca^2+^ levels in Kupffer’s vesicle (an organ in zebrafish that regulates L–R in the heart; Leung et al., 2008). While further studies are needed to characterize this receptor system (i.e., identify downstream signaling pathways), the results of this study indicate that GPR161 may be a therapeutic target for the treatment of congenital heart defects. However the lack of information regarding their endogenous ligands or signaling pathways activated hindered the efforts to identify therapeutics targeting these orphan GPCRs. Advances in homology modeling based on protein crystal structure and *in silico* screening techniques have begun to be applied toward identification of exogenous ligands (to be developed as therapeutics) targeting orphan GPCRs.

## Protein Crystallography

The first reported structure of a GPCR, bovine rhodopsin, was published in 2000 (Palczewski et al., 2000) and was considered a landmark achievement for crystallographers and GPCR biologists. It confirmed that the TM region of GPCRs contains seven α-helices and can serve as a template for other GPCRs, allowing researchers to deduce the location of secondary structural components and highly conserved sequences on related receptors (Palczewski et al., 2000). Successive crystallizations of rhodopsin in various active and inactive conformations with its ligand also provided insights into the mechanisms surrounding GPCR activation (Weis and Kobilka, 2008). Because of the inherent difficulties in crystallizing proteins, it took 7 years for the next GPCR to be crystallized, which was the human β_2_-adrenergic receptor (Cherezov et al., 2007). Since 2007, there have been several breakthroughs in crystallization techniques which have led to a dramatic increase in the number of GPCR structures available. One such technique involves engineering GPCRs to contain small soluble proteins that crystallize under known conditions, in the hope that this will increase the number of lattice contacts necessary for crystal formation between protein molecules (Rosenbaum et al., 2007). This innovative idea was first employed in the crystallization of the β_2_-adrenergic receptor, where the C-terminus and ICL3 of the receptor were replaced with a protein, T4L (Cherezov et al., 2007). A similar strategy was used to crystallize the N/OFQ receptor in 2012 (Thompson et al., 2012), where again the GPCR was modified by fusing a stable protein (in this case, BRIL; Chu et al., 2002) to a truncated N-terminus to facilitate crystal formation (Cherezov et al., 2007). Crystallization has also been accomplished with the help of antibodies, as seen with an additional structure of the β_2_-adrenergic receptor (Rasmussen et al., 2007). Other techniques used to facilitate GPCR crystallization include the use of new detergents and engineering mutations into the receptors (Venkatakrishnan et al., 2013). As a result of these advances, as of January 2015 there are 25 evolutionarily diverse crystal structures of GPCRs available (Classes A, B, C, and Frizzled), spread across various classes (see **Table 1**; Tautermann, 2014). These atomic-level, high-resolution insights into GPCR structure are invaluable in the generation of homology models that will aid in the development of rational, structure-based design of therapeutics targeting GPCRs.

## Protein Structures and Homology Modeling

The modeling process involves several steps beginning with the selection of the protein to be modeled and the template protein, to aligning their primary amino acid sequences and making appropriate corrections, to initial model generation and later refinements, and ending with the validation of the model (Krieger et al., 2003). The most important part of this process is the selection of the appropriate homolog to serve as the template.

Once the protein to be modeled has been chosen (i.e., the target protein) the search for a related protein with known structure (i.e., the template) is accomplished by using sequence-based methods. There are several repositories and structure databases available online, and among them the PDB^1^ is the most popular (Berman et al., 2000). The published structures for proteins are stored at PDB and are easily accessible. Search algorithms such as BLAST (Altschul et al., 1990), compare the entered target’s DNA or amino acid sequence to the sequences of other proteins, identify regions of similarity between them, return a list of proteins ranked in order of their similarity to the query sequence, and also display the alignment (Altschul et al., 1990). Sequences that are more than 40% identical and are found using tools such as BLAST are usually correctly aligned (Saxena et al., 2013), however, errors in the initial alignment are common if the sequences share less than 30% identity (Rost, 1999). It is important to note that BLAST will not work on difficult targets (proteins with only distantly related homologs), and more sensitive method(s) are needed (such as those that compare multiple sequence alignments) in these instances. While it is possible to optimize the initial alignment using specialized alignment software (Nayeem et al., 2006), currently there are no modeling programs available that can construct an accurate model from an incorrect alignment (Sanchez and Sali, 1997) and therefore every attempt should be made to correctly align the target and template sequences.

To model proteins such as GPCRs, it is crucial that there be an adequate supply of highly resolved, diverse GPCR crystal structures that can serve as templates. The advances in GPCR protein crystallization, mentioned previously, have generated a cache of templates for modelers to choose from. Information about the list of published structures is available at http://zhanglab.ccmb.med.umich.edu/GPCR-EXP/ and a visualization of the phylogenetic tree of GPCRs shows the diversity of available structures^2^ (Stevens et al., 2013) and is illustrated in **Table 1**.

## Advances in Homology Modeling of GPCRs

With the increase in the number of templates available for GPCRs there has been renewed interest in improving the methodologies and techniques that are used to create homology models. These include multi-template modeling approaches, MDs, and the treatment of long loop regions in the receptors (Yarnitzky et al., 2010). These are described below.

As its name suggests, multi-template modeling involves the use of more than one template to create a model of the target. The effect of using multiple templates to generate models of the β_2_-adrenergic receptor was evaluated in one paper by comparing the RMSD of ligand poses in multiple and singular template models of the receptor compared to its crystal structure (Mobarec et al., 2009). The multi-template model performed slightly better than the single-template models, since it had a slightly higher percentage of docking poses of the ligand with an RMSD less than 2 Å compared to the crystal structure (Mobarec et al., 2009). However, it should be noted that this improvement was only observed when all the templates used shared low sequence similarity to the target; if the templates had a relatively high sequence similarity to the target, there was no significant difference in the performance of multi-template and single-template models (Mobarec et al., 2009). Another example of successful multi-template modeling is in the enrichment of VLS of homology models of the NK1 receptor (Kneissl et al., 2009). In this study, models of NK1 were built using either bovine rhodopsin or human β_2_-adrenergic receptor as a template, or using a combination of bovine rhodopsin and β_2_-adrenergic receptor as template (Kneissl et al., 2009). NK1 models built based on only one template did not conform to experimental mutagenesis data and needed further refinements; however, the multiple-template model achieved enriched VLS scores in agreement with a consensus model without any additional refinements (Kneissl et al., 2009).

One of the more problematic aspects of modeling GPCRs is in the treatment of variable loop regions. The ECLs and ICLs between GPCRs are much less conserved and often differ between target and template, which makes them extremely difficult to model using template-based approaches (Goldfeld et al., 2011). This is perhaps the greatest disadvantage of using template-based homology models because these loop regions are extremely important in ligand binding and receptor activation (Goldfeld et al., 2011). The ECLs of GPCRs have been shown to facilitate the binding of ligands of several different chemotypes including small drug-like molecules (de Graaf et al., 2008), large proteins (de Graaf et al., 2008), and various low molecular weight biological compounds (Goldfeld et al., 2011) and can also regulate receptor activation (Klco et al., 2005). ICLs group together to form functional domains that interact with the G-proteins coupled to the receptor (Wong, 2003). Because these regions are so integral to proper GPCR function much effort has gone into improving the modeling of these unpredictable peptide sequences, and what to do if loop modeling proves too complex. For this the modeling field has moved toward more template-free approaches that use energy functions to guide loop folding in a defined conformational space (Li, 2013). During this process a large number of models are generated and the best models are chosen for further refinement based on their minimized energy in terms of geometric constraints (i.e., minimized steric constraints, etc.; Li, 2013). The success of this method relies on the ability of algorithms to effectively sample the best conformations and evaluate model energy (Tang et al., 2014). Since 2000 several programs have been developed for loop modeling and each uses different energy functions and sampling criteria (Li, 2013). Some popular programs among many include MODELLER (uses statistical potentials to integrate restraints or pseudo-energy; Fiser et al., 2000), LOOPY (uses colony energy; Xiang et al., 2002), RAPPER (uses AMBER force field combined with the Generalized Born solvation model; de Bakker et al., 2003), Rosetta (uses Rosetta; Rohl et al., 2004), and Looper (uses CHARMM force field with additional parameters; Spassov et al., 2008). In general these programs are very effective at modeling loops as long as eight residues (Li, 2013), and newer methods can accurately predict loop structures up to 13 residues long (Mandell et al., 2009; Li et al., 2011), but modeling longer loops still remains a significant challenge (Li, 2013).

In recent years homology models of orphan GPCRs have been generated. For example, a model for GPR18, a Class A receptor that might play a role in apoptosis, using the N/OFQ receptor as a template has been generated (Kothandan and Cho, 2013). Also two homology models were generated to examine the effects of mutations on the structure of the P2RY5 receptor, an orphan GPCR identified by genetic linkage analysis that is implicated in autosomal recessive wooly hair (Shimomura et al., 2008). In addition, a homology model of GPR55 was built using the crystal structure of the adenosine A (2A) receptor and used to dock known ligands (Elbegdorj et al., 2013).

Molecular dynamic simulations are time-dependent computer-based simulations that track the movement of atoms and molecules in a defined system (Lindahl, 2008). When MDs are applied to large proteins such as GPCRs they can reveal important structural information, and in general serve as a means to assess how theoretical models (such as homology models) compare to experimental data (Lindahl, 2008). In the case of GPCRs, MDs are recorded on a timescale ranging from nanoseconds to microseconds and can be used to study not only how a ligand reaches the binding pocket of a GPCR but also the mechanism of receptor activation, oligomerization, and allosteric modulation (Bruno and Costantino, 2012). In homology modeling MDs are used for further refinement to optimize protein folding (Yarnitzky et al., 2010) thereby improving the performance of the model for ligand docking simulations (Yarnitzky et al., 2010). In one study bovine rhodopsin was used as a template to create a model of the HH4R, and MDs were run on the receptor in a membrane-bound environment with and without its agonist histamine and a selective antagonist (Jójárt et al., 2008). The simulations modeled receptor conformational changes and histamine-binding site interactions that agreed with previous experimental data (Jójárt et al., 2008). In addition, the modeled movements of several TMs were also in agreement with experimental data. Taken together these results indicated that the MDs of the HH4R can serve as a structural model for identification of HH4R ligands (Jójárt et al., 2008).

Molecular dynamic simulations can also be employed in an innovative way to address some problems associated with ligand binding to homolog During these MDs, pressure is applied to the binding site of the receptor to expand it much in the same way that a balloon expands when filled with air and for this reason this is sometimes called the “balloon expansion method” (Kimura et al., 2008). To test the legitimacy of this method, a structure of bovine rhodopsin was created without its ligand retinal, rendering it inactive and closed. Upon application of the “balloon pressure” retinal was able to bind almost exactly as it is known to (Kimura et al., 2008). When tested on an unrefined homology model of the chemokine receptor CCR2 that was originally unable to bind three known antagonists, the applied pressure allowed for the ligands to bind in a manner that agreed with mutagenesis data (Kimura et al., 2008).

## *In Silico* Screening Using Homology Models of GPCRs for Drug Discovery

One of the most practical and popular applications of homology models is their use in *in silico* virtual screens for drug discovery. An example of successful use of homology modeling to identify small molecule ligands is that of DRD3. In this case, a homology model of the DRD3, built using the β_2_-adrenergic receptor as a template, was used to dock over 3 million compounds (Carlsson et al., 2011). When the 26 top-scoring molecules were tested for binding affinity, six of these compounds exhibited affinities in the micromolar range (Carlsson et al., 2011). Soon after this initial screen, the crystal structure for the DRD3 receptor was released and the same docking experiment was performed; this yielded five molecules with affinities between nanomolar and micromolar concentrations (Carlsson et al., 2011). The hit rates between model and crystal structure were also similar, at 23 and 20%, respectively. What is remarkable about this study is that there was no difference in the screening capability between the homology model and crystal structure; the model was able to identify binding ligands at the same rate as the crystal structure (Carlsson et al., 2011). While this study demonstrates the effectiveness of homology models in virtual screens, this was not the first time that homology models were successfully used to predict novel ligands for GPCRs. Homology models of the HH4R (Kiss et al., 2008), FFAR1 (Tikhonova et al., 2008), and MCHR1 (Cavasotto et al., 2008), among other examples (Edwards et al., 2005; Engel et al., 2008) were able to identify novel binding compounds that exhibited either agonistic or antagonistic properties (**Table 2**). A recent review (Kooistra et al., 2013) lists over 15 examples of successful structure-based virtual screens of GPCR homology models (**Table 2** summarizes six of these virtual screens). The success with the identification of these ligands clearly demonstrates the usefulness of homology models in ligand discovery. Virtual docking approaches similar to those used in the case of models of known GPCRs are likely to be useful in the identification of ligands targeting orphan GPCRs.

**Table 2:** Novel ligands predicted by virtual screening against GPCR homology models.

Receptor	Ligand with highest affinity	Agonist or antagonist	Reference
			


DRD3		Antagonist *K*_i_ = 0.3 μM	Carlsson et al. (2011)


	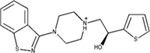		
			


HH4R		Antagonist *K*_i_ = 85 nM	Kiss et al. (2008)


	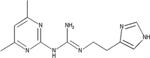		
FFAR1		Agonist *EC*_50_ = 3.6 μM	Tikhonova et al. (2008)


	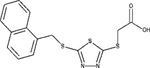		
MCHR1		Antagonist *K*_i_ = 7.5 μM	Cavasotto et al. (2008)


	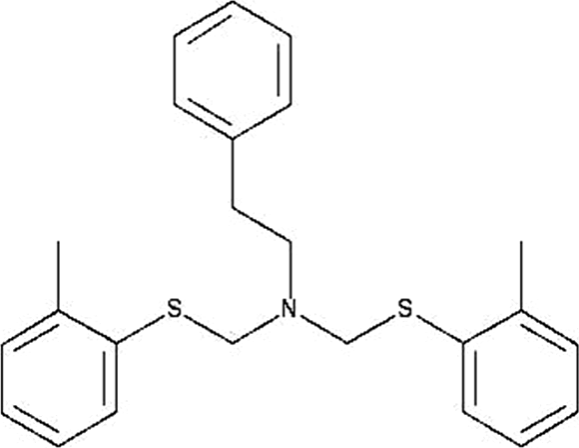		
FPR1R		Partial agonist *K*_i_ = l μM	Edwards et al. (2005)


	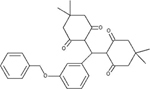		
TRHR		Antagonist *K*_i_ = 0.29 μM	Engel et al. (2008)


	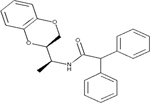		

As described above, while the GPCR homology models have been used to study structural features of orphan receptors, homology modeling has not yet been widely applied in the context of *in silico* drug screens. Perhaps the best example of a successful *in silico* compound screen for an orphan receptor is in the case of GPR17. A homology model of the receptor was created using the multi-template approach by combining the crystal structures of four GPCRs, and was then used to screen an in-house chemical library (the Asinex Platinum Collection) containing 130,000 lead-like compounds (Eberini et al., 2011). The binding site of the receptor was identified using the MOE Site Finder module, which evaluates possible binding sites according to their geometry and location; it is a ligand-free approach and does not use known binding compounds to detect the binding site (Eberini et al., 2011). The top-5 scoring compounds (compounds binding with the lowest energy conformations) were purchased and receptor activation was measured in dose-response [^35^S] GTPγS binding assays (Eberini et al., 2011). Four of these compounds displayed full agonist activity and one compound was shown to act as a partial agonist, as compared to a reference ligand; all compounds exhibited nanomolar or sub-nanomolar potencies (Eberini et al., 2011). This virtual screen was also able to identify efficacious molecules with novel chemical scaffolds; these compounds are the first examples of GPR17 ligands that are not derived from the structures of previously known GPR17 activators (i.e., CysLTR1 or P_2_YR ligands; Eberini et al., 2011). While GPR17 is not a strictly orphan receptor, almost no information regarding known ligands was needed to execute this study (except for activity characterization/comparison in the *in vitro* experiments) and it therefore serves as a good example of novel and efficacious lead-compound discovery at a virtually unknown receptor system.

## Conclusion

GPCRs are the most targeted sites for drug discovery and this trend is not predicted to end soon. Moreover, breakthroughs in protein crystallization strategies and computer technology have opened the doors for *in silico* methods of studying these receptors. This includes advances in GPCR crystallization that have led to the release of many new crystal structures, igniting renewed interest in the structure-based study of GPCRs. Thus GPCR homology modelers in need of templates now have dozens of functionally and evolutionary diverse options to choose from. This taken together with the recent advances in the computational aspects of modeling make GPCR homology models more accurate. As a result, virtual ligand screens using these models have successfully identified both known ligands and novel compounds. The performance of GPCR homology models in virtual screens compared to crystal structures is impressive, and clearly validates their continued use in ligand discovery. This bodes well for the homology modeling of orphan GPCRs and the use of modern *in silico* screening methods to identify putative ligands, both agonists and antagonists that could be used to elucidate the physiological role of the receptor.

The combination of computational and biological methods provides a unique approach to studying orphan GPCRs that offers several advantages over strictly biological approaches. Thus if computational methods are applied to orphan receptors, they provide an unbiased approach at ligand discovery which enhances the chances for successful discovery of novel compounds, and novel chemical scaffolds. Moreover, while biological methods are limited by the amount of resources (cells, compounds, etc.) available for receptor ligand screening, *in silico* screening allows for the testing of 1000s of compounds without costly *in vivo* or *in vitro* screening. As it stands, ligand discovery for orphan GPCRs via computational methods and validation of lead compounds is ripe for exploration, and stands to be at the cutting edge of orphan GPCR research.

## Conflict of Interest Statement

The authors declare that the research was conducted in the absence of any commercial or financial relationships that could be construed as a potential conflict of interest.

## Footnotes

1 http://www.rcsb.org

2 http://gpcr.scripps.edu/

## Abbreviations

BLAST: basic local alignment search tool
BRIL: apocytochrome b562RIL
cAMP: cyclic AMP
CCR2: C-C chemokine receptor type 2
CysLTR1: cysteinyl leukotriene receptor 1
DAG: diacylglycerol
DARPP-32: dopamine- and cAMP-regulated phosphoprotein of 32 kDa
DRD3: dopamine D_3_ receptor
ECL: extracellular loop
FFAR1: free fatty acid receptor 1
FPR1R: formylpeptide receptor
GABA: gamma-aminobutyric acid
GHS-R: growth hormone secretagogue receptor
GPCR: G-protein coupled receptor
GRAFS: glutamate, rhodopsin, adhesion, frizzled/taste2, secretin
H: helix
HH4R: human histamine H4 receptor
ICL: intracellular loop
Ins(1,4,5)P3: inositol 1,4,5-trisphosphate
KD/KO: knockdown/knockout
MCHR1: melanin-concentrating hormone receptor 1
MDs: molecular dynamic simulations
NK1: neurokinin 1 receptor
N/OFQ: nociception/orphanin FQ receptor
ORL-1: opioid receptor-like 1
OX_1_R: orexin receptor type 1
OX_2_R: orexin receptor type 2
P2RY5: purinergic receptor 5
PLCβ: phospholipase Cβ
PDB: protein data bank
PKA: protein kinase A
PKC: protein kinase C
RMSD: root-mean square deviation
T4L: T4-lysozyme
TM: transmembrane
TRHR: thyrotropin-releasing hormone receptor
VLS: virtual ligand screening.
